# Prevalence and predictors of long-term progression of chronic kidney disease in people with HIV in Ghana from 2003–2018

**DOI:** 10.1186/s12882-024-03537-7

**Published:** 2024-07-29

**Authors:** David R. Chadwick, Fred Barker, Colette Smith, Okyere Perditer, Yasmine Hardy, Dorcas Owusu, Giovanni Villa, Fred Stephen Sarfo, Anna-Maria Geretti, Richard Phillips

**Affiliations:** 1 Centre for Clinical Infection, James Cook University Hospital, Middlesbrough, TS4 3BW UK; 2 Tyne and Wear, Northumbria Healthcare Trust, North Shields, NE29 8NH UK; 3 Institute for Global Health, University College London, London, NW3 2PF UK; 4 Department of Medicine, Komfo Anokye Teaching Hospital, 1934 Adum-Kumasi, Kumasi, Ghana; 5 School of Medicine and Dentistry, Kumasi Centre for Collaborative Research in Tropical Medicine, Kwame Nkrumah University of Science & Technology, Kumasi, Ghana; 6 Department of Genitourinary Medicine and Infectious Diseases (GUIDe Clinic), St. James’ Hospital, Dublin, Ireland; 7 School of Immunology & Microbial Sciences, King’s College London, London, SE5 9RS UK; 8 Tor Vergata University of Rome, 50, 00133 Rome, Italy

**Keywords:** HIV, Kidney, Renal, Chronic kidney disease, Africa, Antiretroviral therapy

## Abstract

**Background:**

HIV is associated with an increased risk of progression to chronic kidney disease (CKD), and this risk is higher in people of West African descent than many other ethnicities. Our study assessed the rates of eGFR change and predictors of rapid eGFR progression in patients receiving antiretroviral therapy (ART), including tenofovir disoproxil fumarate (TDF), in central Ghana between 2003 and 2018.

**Methods:**

This single-centre retrospective study enrolled people with HIV (PWH) initiating ART in Ghana between 2003–2018. Demographics, hepatitis B (HBsAg) status, ART regimens and estimated glomerular filtration rate (eGFR) measurements were recorded, and analyses including multi-level model linear regression were performed to determine predictors of greater levels of eGFR decline and risk of rapid eGFR decline.

**Results:**

Six hundred and fifty-nine adult participants were included in the study with a median follow-up time of 6 years (IQR 3.6–8.9). 149 participants (22.6%) also had confirmed HBV co-infection. eGFR mean values were lowest at the point of diagnosis and highest on the second measurement taken; mean eGFR slowly decreased over subsequent measures thereafter. TDF use was associated with the highest mean rate of eGFR decline of all nucleoside or nucleotide reverse transcriptase inhibitors (NRTIs) with a statistically significant greater annual decline of -1.08 mL/min/1.73m^2^/year (CI: -1.92, -0.24) compared with zidovudine. Nevirapine (-0.78mL /min/173m^2^/year; CI: -1.39, -0.17) and protease inhibitors (-1.55mL/mil/173m^2^/year; CI: -2.68, -0.41) were associated with greater eGFR declines compared with efavirenz. Negative HBsAg status was associated with greater eGFR decline compared with positive HBsAg status (-1.25mL/mil/173m^2^/year; CI 0.29. -2.20).

**Conclusions:**

Increased rates of eGFR decline amongst PWH in Ghana were associated with TDF, nevirapine, and protease inhibitor use as well as negative HBsAg status. Additional research using mortality outcome data is needed to closely assess long-term predictors of eGFR decline in African populations.

## Background

People with HIV (PWH) are at increased risk of chronic kidney disease (CKD) secondary to disorders including HIV-associated nephropathy (HIVAN), HIV immune complex glomerulonephritis, diabetes and hypertension. Antiretroviral treatments (ART) for HIV have also been shown to adversely affect renal function in PWH [1–9], though on balance the use of ART has considerably decreased both HIV-associated CKD and progression to end stage renal disease (ESRD) [10-13]. However, renal disease remains common amongst PWH and those affected have higher mortality risk than those with good renal function [14, 15].

Africa has the highest prevalence of HIV-associated CKD globally. West Africa is the most affected region in Africa with a prevalence (15%) five times greater than that of southern Africa [16]. Amongst PWH of African ethnicity in the UK, West Africans have also been found to be at highest risk of ESRD [17]. On the African continent, major factors contributing to CKD progression include genetic predisposition [18–21] and co-infections such as hepatitis B virus (HBV) [22], which affects approximately 14%-17% of PWH in Ghana [23, 24]. Studies have noted that chronic HBV infection has associations with CKD or progression to CKD both in PWH [22, 25] and other populations [26]. However, there is a paucity of long-term research exploring this relationship in African co-infected populations, with only one study evaluating it specifically as a risk factor for CKD progression [27].

The nucleotide reverse transcriptase inhibitor TDF is active against both HIV and HBV. It has been frequently and widely used in Sub-Saharan Africa as a first-line ART for over a decade. Despite modest evidence of favourable side effect profiles over some other common antiretrovirals [28, 29], TDF has demonstrated associations with impaired glomerular filtration and tubular dysfunction (including Fanconi’s syndrome) in both HIV positive and negative populations [30–34]. A systematic review of African studies showed statistically significant positive relationships between TDF use and renal dysfunction in around a quarter of its included studies [35]. However, few published studies have assessed how renal function changes over longer periods of time [36–38]. One of these studies noted a significantly greater eGFR decline in the TDF group compared with the control [37].

Our single-centre study was designed to determine risk factors associated with worsening eGFR or developing fast eGFR decline in Ghanaian PWH taking ART over up to a 15-year period. Our specific focus was the influence of individual ART drugs and HBV co-infection on renal function decline.

## Methods

### Setting

The study was conducted at the Komfo Anokye Teaching Hospital (KATH) in Kumasi, Ghana. The Committee on Human Research Publications and Ethics at Kwame Nkrumah University of Science and Technology (KNUST) approved this study. All PWH provided written consent to take part in the study.

### Study population and therapy

The majority of participants were enrolled from previous studies with retrospective data collection starting in 2004 and completed in 2019 [14, 27, 39]. In addition, a small convenience sample of PWH attending the clinic in February 2019 were enrolled explicitly for this study, where retrospective data were collected from point of HIV diagnosis up to February 2019 and typically had a year or more between each measurement. The study included adults (18 years or older) who had both a baseline creatinine measurement (within 3 months of starting ART) and at least one further result beyond this date.

National HIV treatment guidelines (2003) originally recommended that PWH were initiated on either zidovudine or stavudine with lamivudine, plus either nevirapine or efavirenz, with some protease inhibitors (PI), didanosine and abacavir available for second-line therapy. Once TDF became available in 2010, a small proportion of patients, including most of those identified with hepatitis B co-infection, were commenced on or switched to TDF.

### Data collection and definitions

All demographic, clinical and laboratory data used were collected from clinic case notes or research registers. Participants’ first recorded ART regimens were used to define their ‘baseline’ regimen. Data were recorded according to the type of ART used and the length of time it was taken for. Cases where a patient switched between different ART medications were recorded for later data analysis.

The creatinine data used in this study was mostly from samples as part of routine or standard hospital care (locally measured), or from one of several research studies (measured in the UK). CKD stages 1, 2 and 3 were defined by a single eGFR measurement of ≥ 90, 89.9–60 and < 60, respectively. Routine management of PWH in KATH did not include regular blood creatinine measures, rather these were done periodically or when felt to be clinically indicated. Likewise, until recently most PWH were not routinely tested for hepatitis B surface antigen (HBsAg) unless they were found to have deranged transaminase measurements. None-the-less, a significant number of patients attending the clinic were tested for HBsAg and had more frequent creatinine measurements as part of previous studies. HIV viral load is also not routinely measured and was not included in any analyses.

PWH were coded as hepatitis B co-infected if they had at least one positive HBsAg test recorded. eGFR was calculated using the CKD-EPI creatinine equation with standard adjustment for gender and age. In line with previous analyses of HIV-positive populations [40], rapid eGFR progression was defined as a change in eGFR of greater than 5 ml/min/1.73m^2^ per year between baseline and final eGFR measurement.

### Statistical analysis

Two study populations were considered for analysis: (i) the sub-set of 545 participants with at least three years’ follow-up, chosen to ensure medium-to-long-term eGFR changes were being evaluated; (ii) all 659 participants with at least two eGFR measurements for formal regression analysis, to maximise the use of available data as well as to minimise risk of bias associated with loss-to-follow-up. Among the 545 participants with at least 3 years’ follow-up, the annualised eGFR change (the difference between the first and last eGFR measurements, divided by the length of follow-up in years) was calculated and compared according to antiretroviral use and hepatitis B (HBsAg) status using analysis of variance (ANOVA). In addition, the percentage that had experienced rapid progression over > 3 years’ follow-up, as defined above, was calculated stratified by hepatitis B and ART status and compared using a chi-squared test. Next, considering all 659 participants, a multi-level model was constructed to investigate factors associated with eGFR changes over time. Time was considered as a linear association. Although covariates were also included as main effects, the results focus on the association between covariates and the rate of eGFR change over time (that is the covariate-time interaction terms). An unstructured correlation matrix was used, and random effects terms were included for the intercept were performed using SAS Version 9.4 (SAS Institute Inc, Cary, NC).

### Sensitivity analysis

Sensitivity analysis assessed whether results remained consistent in a restricted cohort where only participants with at least three years between their first and last eGFR result were included.

## Results

### Study population characteristics and baseline eGFR

After review of research databases (*n* = 463) and the convenience sample (*n* = 196), 659 PWH on ART were identified with two or more eGFR measurements available; 545 of these had at least 3 years between first and final measures. The characteristics of this population are described in Table 1.

**Table 1:** Patient characteristics at start of antiretroviral therapy

	**N (%), mean (SD) or median [IQR; range]**	
	**All participants**	**Those with > 3 years’ follow-up**
N	659	545
Gender		
Female	488 (74.1%)	404 (74.1%) 141 (25.9%)
Male	171 (26.0%)	
Age (years)	42 [25, 48; 20 77]	42 [36, 49; 20, 77]
Baseline CD4 count (cells/microL)	201 (163.5)	203 (161.3)
Baseline eGFR (ml/min/1.73m^2^)	104.5 (31.4)	103.7 (31.1)
Hepatitis B status		
Positive	149 (22.6%)	96 (17.6%)
Negative	210 (31.9%)	165 (30.3%)
Not known	300 (45.5%)	284 (52.1%)
NRTI combination at baseline		
Zidovudine based	391 (59.3%)	343 (62.9%)
Stavudine based	183 (27.8%)	149 (27.3%)
TDF based	77 (11.7%)	46 (8.4%)
Other	8 (1.2%)	7 (1.3%)
‘Third’ antiretroviral at baseline		
Efavirenz	391 (59.3%)	321 (58.9%)
Nevirapine	236 (35.8%)	198 (26.3%)
P	32 (4.9%)	26 (4.8%)
Total follow up time (years)	6.0 [IQR 3.6, 8.9; range 0.0, 16.6]	6.8 [IQR 5.1, 10.0; range 3.0, 16.6]
Number of eGFR measurements available		
2	302 (45.8%)	206 (37.8%)
3	210 (31.9%)	196 (37.0%)
4	147 (22.4%)	143 (26.2%)

The median time between first and final creatinine measurement for the combined cohort was 6 years (IQR 3.6–8.9). For the convenience sample this period was 5 years (IQR 2–6.1). 149 participants (22.6%) had confirmed HBV co-infection (HBsAg positive), 210 (31.9%) were HBsAg-negative and the remainder had unknown HBV status. Most participants started with a zidovudine-based ART (59.3%), while 77 were initiated on Tenofovir disoproxil fumarate-based ART (TDF) (11.7%). Efavirenz was the most common initial 'third’ ART (59.3%).

**Fig. 1:**
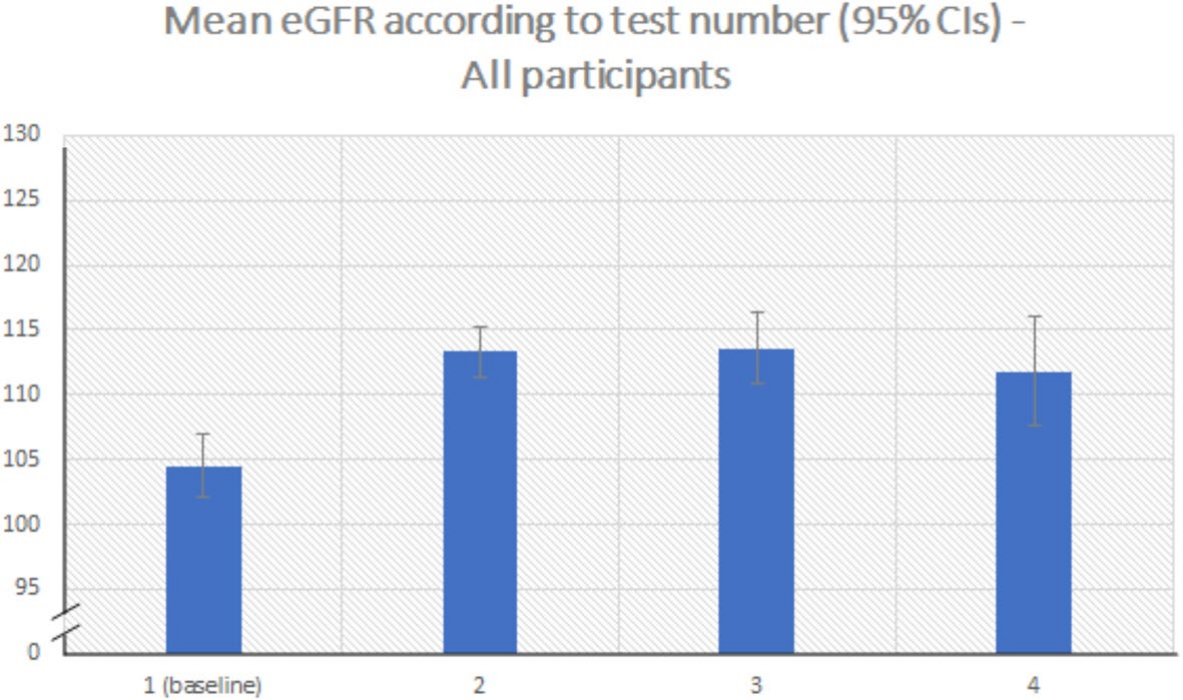
Mean eGFR scores of all participants for baseline, second, third and fourth blood tests. A bar graph was selected over a line graph because time between measures is not accounted for in this graph

At baseline, the mean eGFR of participants was 104.5 ml/min/1.73m^2^ (SD 31.4; range 11.9 to 185.2). 226 participants (34.3%) had an eGFR < 90 ml/min/1.73m^2^ at this stage; 55 (8.4%) had an eGFR < 60 ml/min/1.73m^2^. There was a notable increase in participants’ mean eGFR between first and second measurements, followed by a modest decrease over subsequent measurements (see Fig. 1).

### Associations with eGFR change over time

In the sub-group with at least three years of follow-up, the mean absolute increase in eGFR over the course of the study equated to an annualised value of 1.1 ml/min/1.73m^2^ per year (95% CI 0.6, 1.6; range -27.4, 29.6) (see Table 2). There was considerable variability between individuals, with a standard deviation of 5.9 ml/min/1.73m^2^ per year.

**Table 2:** Evaluation of eGFR among those with at least 3 years’ follow-up, according to ART regimen and HBsAg status

	**N**	**Mean (SD)**
**Absolute change between first and last creatinine measurement (μmol/L)**	545	-12.3 (34.9)
**Absolute change between first and last eGFR measurement (ml/min/1.73m** ^**2**^ **)**	545	+ 6.8 (33.2)
**Years between first and last measurements** ^**a**^	545	6.8 [5.1, 10.0]
**Annualized change in eGFR (ml/min/1.73m** ^**2**^ **)**	545	+ 1.1 (5.9)
**Rapid CKD progression**	545	29 (5.3%)
**eGFR at final measurement** ^**b**^		
eGFR ≥ 90 (Cutoff for CKD stage 1)	545	430 (65.3%)
eGFR 60–89.9 (Cutoff for CKD stage 2)		96 (14.6%)
eGFR < 60 (Cutoff for CKD stage 3)		19 (2.9%)
Annualised eGFR change according to characteristics **(ml/min/1.73m** ^**2**^ **/year)**:		
**By hepatitis B status (** ***p*** ** < 0.0001)**		
Positive	96	+ 1.1 (5.9)
Negative	165	-1.0 (3.9)
Unknown	284	+ 2.3 (6.6)
** By baseline NRTI (** ***p*** **< 0.0001)**		
Zidovudine based	343	+ 0.5 (5.3)
Stavudine based	149	+ 3.0 (7.1)
TDF based	46	-0.7 (4.8)
Other	7	+ 0.3 (8.0)
** By baseline PI/NNRTI (ml/min/1.73m** ^**2**^ **) (** ***p*** ** = 0.22)**		
Efavirenz	321	+ 1.4 (5.8)
NVP	198	+ 0.8 (6.2)
PI	26	-0.4 (5.5)
** By NRTI use over follow-up (** ***p*** **< 0.0001)**		
Remain on zidovudine	308	+ 0.7 (5.3)
Zidovudine/stavudine/other switch to TDF	46	-0.8 (4.8)
Non-TDF switch	64	+ 1.6 (5.4)
Remain on stavudine	75	+ 4.6 (8.1)
TDF switch to zidovudine/ stavudine/other	1	+ 5.4
Remain on TDF	45	-0.8 (4.7)
Remain on other	6	+ 0.4 (8.7)
** By PI/NNRTI use over follow-up (** ***p*** ** = 0.087)**		
Remain on efavirenz	300	+ 1.5 (5.8)
Switch between NNRTIs	29	-1.2 (4.6)
Switch from NNRTI to p	19	+ 0.3 (4.6)
Remain on NVP	171	+ 1.0 (6.4)
Switch from PI to NNRTI	1	-7.4
Remain on PI	25	-1.6 (5.4)

HBsAg – hepatitis B surface antigen

^a^Median and inter-quartile range

^b^number and percentage

Significant discrepancies in eGFR changes were noted within hepatitis B status subgroups (*p* < 0.0001), with eGFR increases seen among those of unknown status or HBsAg positive individuals but net eGFR decline over time for the HBsAg negative group. There were also significant differences in eGFR over time for NRTI drug subgroups, with greatest eGFR increases in those receiving stavudine (d4T) based NRTI backbones. There was no evidence of an association with specific NNRTI or protease inhibitor use.

Multi-level models considering all 659 participants identified that treatment with TDF was associated with the poorest eGFR outcomes of all NRTIs (Table 3), with a statistically significant eGFR greater decline per year of –1.08ml/min/1.73 m^2^ (CI –1.92, -0.24) compared with zidovudine. TDF was also the only drug which was associated with a net negative change in eGFR irrespective of whether it was commenced from HIV diagnosis or switched onto from a different NRTI (Table 2). In contrast, stavudine was associated with significantly favourable eGFR outcomes, with an average change per year that was + 1.21ml/min/1.73 m^2^ (95% CI + 0.25, + 2.16) greater compared with zidovudine. The analysis found no significant difference in relative eGFR change over time between zidovudine and pooled results from the remaining less common NRTIs (difference –1.53 ml/min/1.73 m^2^, 95% CIs –0.92, 3.99). There was evidence that NVP and PI use were associated with less favourable eGFR changes over time compared to efavirenz, and that positive and unknown HBsAg status were associated with favourable eGFR changes over time compared to HBsAg negative status.

**Table 3:** Factors associated with eGFR change per year: results from multi-level model incorporating all eGFR measurements recorded over a period up to 15 years

		**Univariable**			**Multivariable**		
		**Beta**	**95% CI**	**P**	**Beta**	**95% CI**	**P**
Gender (vs male)	Female	-0.47	-1.10, 0.17	0.15	-0.31	-0.96, 0.33	0.35
Baseline age	Per 10 years	-0.17	-0.46, 0.12	0.25	0.13	-0.19, 0.44	0.43
Current NRTI (vs zidovudine)	Stavudine	1.69	0.73, 2.66	< 0.0001	1.21	0.25, 2.16	0.008
	TDF	-1.60	-2.39, -0.80		-1.08	-1.92, -0.24	
	Other	0.69	-1.54, 2.92		1.53	-0.92, 3.99	
Current Third drug (vs efavirenz)	NVP	-0.77	-1.38, -0.16	0.0009	-0.78	-1.39, -0.17	0.0029
	PI	-1.73	-2.76, -0.70		-1.55	-2.68,-0.41	
Hepatitis B (vs Negative)	Positive	1.51	0.56, 2.46	< 0.0001	1.25	0.29, 2.20	< 0.0001
	Unknown	1.65	1.07, 2.24		1.38	0.74, 2.01	

### Sensitivity analysis

Sensitivity analysis considering the multi-level model restricted to the sub-group with longer follow up found consistent results with the main findings (Table 4).

**Table 4:** Multivariable sensitivity analysis where only participants with follow-up period spanning > 3 years were included in analysis

		**Univariable**			**Multivariable**		
		**Beta**	**95% CI**	**P**	**Beta**	**95% CI**	**P**
Gender (vs male)	Female	-0.38	-1.04, 0.27	0.25	-0.32	-0.98, 0.34	0.34
Baseline age	Per 10 years	-0.18	-0.47, 0.12	0.23	0.11	-0.21, 0.43	0.51
Current NRTI (vs zidovudine)	Stavudine	1.67	0.70, 2.65	< 0.0001	1.20	0.23, 2.16	0.004
	TDF	-1.46	-2.31, -0.61		-1.00	-1.90, -0.11	
	Other	0.22	-2.04, 2.49		1.06	-0.44, 3.56	
Current Third drug (vs efavirenz)	NVP	-0.77	-1.39, -0.15	0.0005	-0.73	-1.35, -0.10	0.005
	PI	-1.90	-2.95, -0.84		-1.60	-2.78,-0.41	
Hepatitis B (vs Negative)	Positive	1.27	0.29, 2.26	< 0.0001	1.07	0.07, 2.06	0.0003
	Unknown	1.48	0.89, 2.08		1.32	0.66, 1.96	

### Prevalence of rapid eGFR progression

Table 5 provides information on the proportion of participants in each subgroup who demonstrated rapid eGFR deterioration in the sub-group with > 3 years’ follow-up. In total, 5.3% (29/545) experienced an annualised decline in eGFR of > 5 ml/min/1.73m^2^/year, as well as a final eGFR value < 90 ml/min/1.73m^2^. There were statistically significant differences in risk to progression according to ‘third’ ART choice (*p* = 0.0013), though no statistically significant differences within groups according to HBV status.

**Table 5:** Frequency of Rapid progression (RP) during study period, according to ARV regimen and HBV statusv

	**n/N (%)**	***p*** **-value**
Total	29/545 (5.3%)	
**HBV status**		
Positive	6/96 (6.3%)	0.89
Negative	9/165 (5.5%)	
Unknown	14/284 (4.9%)	
**Baseline NRTI**		
Zidovudine based	21/343 (6.1%)	0.55
Stavudine based	5/149 (3.4%)	
TDF based	3/46 (6.5%)	
Other	0/7 (0.0%)	
**PI/NNRTI at baseline**		
Efavirenz	16/321 (5.0%)	0.35
NVP	10/198 (5.1%)	
PI	3/26 (11.5%)	
**NNRTI at both measurements**		
Remain on zidovudine	16/308 (5.2%)	0.61
Zidovudine/ Stavudine /other switch to TDF	5/46 (10.9%)	
Non-TDF switch	3/64 (4.7%)	
Remain on stavudine	2/75 (2.7%)	
TDF switch to zidovudine/stavudine/Other	0/1 (0.0%)	
Remain on TDF	3/45 (6.7%)	
Remain on Other	0/6 (0.0%)	
**PI/NN at each measurement**		
Remain on efavirenz	14/300 (4.7%)	0.0013
Switch between NNRTIs	3/29 (10.3%)	
Switch from NNRTI to PI	1/19 (5.3%)	
Remain on NVP	8/171 (4.7%)	
Switch from PI to NNRTI	1/1 (100.0%)	
Remain on PI	2/25 (4.0%)	

## Discussion

Our findings provide evidence that many PWH in Ghana see an initial increase in eGFR over their first years of taking ART. Participants in this study demonstrated an initial increase in mean eGFR which was largely sustained over the study period; findings such as these have been seen from previous studies on similar populations [41, 42]. These results likely reflect a combination of factors relating to studying a population with considerably higher rates of advanced HIV infection (and HIV-associated nephropathies) at diagnosis compared to high-income country cohorts. One factor might be due to many participants being acutely unwell at diagnosis, then improving significantly following treatment; once ART was commenced and they clinically improved, renal function may have returned closer to their baseline level. Factors such as survivor bias may have also artificially ‘boosted’ mean eGFR changes if PWH with poorer outcomes did not survive and their data were missed.

Our results support previous findings highlighting that TDF and protease inhibitors have greater associations with eGFR decline compared with other antiretroviral drugs [27, 32–35]. Nevirapine also showed significantly greater associations with eGFR decline compared to efavirenz and there were significant differences in proportions of participants who experienced rapid eGFR decline.

It is important to note that in spite of the overall increase in eGFR across our participants, TDF was the only drug noted to still demonstrate negative associations with eGFR decline irrespective of whether it was started at baseline or if switched to at a later date. This strongly supports the case for more rapidly switching ART regimens to include tenofovir alafenamide (TAF), and integrase inhibitors, or tenofovir-sparing regimens such as dolutegravir/lamivudine (in non-HBV co-infection), which are now more widely available in Ghana and sub-Saharan Africa.

**Fig. 2:**
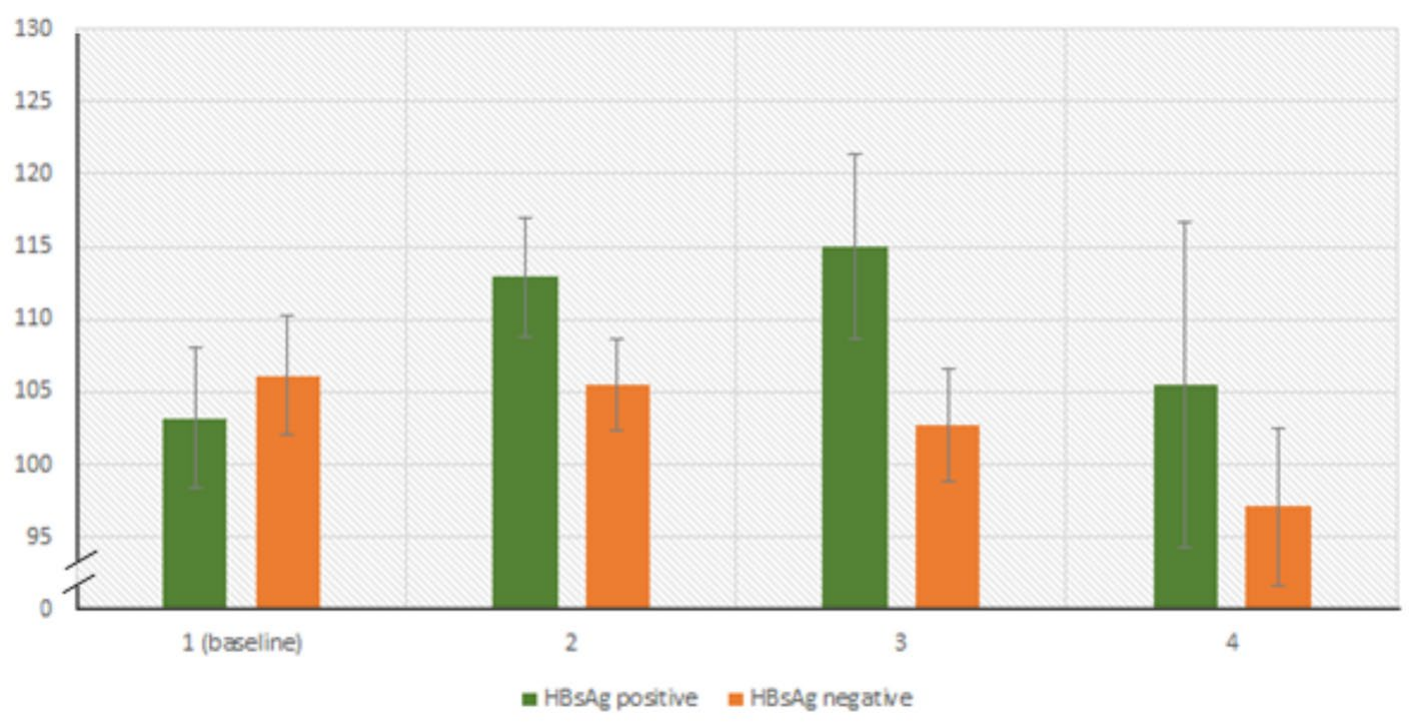
Post-hoc analysis of mean eGFR scores in HBsAg positive and negative participants for baseline, second, third and fourth bloods tests

The finding of a positive effect of HBsAg co-infection on eGFR outcomes was unexpected based on our initial hypotheses and is not consistent with evidence from the literature assessing HBV’s association with CKD [43]. Post-hoc analysis of our results highlighted a mean eGFR increase of almost 10 ml/min/1.73m^2^ between first and second blood tests in HBV positive participants, but no increases in HBV negative participants (Fig. 2). This is particularly interesting because HBsAg positive patients in this cohort were considerably more likely to have taken TDF, which itself was associated with the least favourable outcomes of all NRTIs. The possibility exists that HBsAg positive patients were further off their eGFR baseline at point of diagnosis due to comorbidity, which saw a large improvement in function following ART. Nonetheless, we believe sampling bias likely also influenced these results. HBV testing has not historically been routine practice in HIV management in Ghana, so a main driving factor for HBsAg tests being requested in PWH (outside of research studies) was when participants had elevated transaminase enzymes. As a result, patients with tested elevated transaminase enzymes (but later shown to be HBsAg negative) may have had alternative comorbidities with more unfavourable associations with eGFR decline. Participants with ‘unknown’ HBsAg function had not been tested in most cases, and likely have had differences in their overall care journey compared with those tested for HBsAg, leading to a different mean score.

### Limitations

Our study cohort was relatively young, with less than a quarter of participants being above 50 years old. There is some evidence that renal function may decline at faster rates as people age [44], which suggests our measures of eGFR decline may not reflect real-world rates in older PWH. A younger cohort is also likely to underestimate incidence of CKD development as participants’ baseline kidney functions are more frequently well above CKD-relevant eGFR thresholds.

While our definition of CKD used identical CKD eGFR cut-offs as the standard’Kidney Disease: Improving Global Outcomes’ (KDIGO) criteria [45], the limited number of creatinine measurements per participant meant only a single eGFR measurement defined CKD stages (deviating from KDIGO’s requirement of a sustained eGFR value for three months). However, as our primary outcome variable was eGFR change, this did not significantly affect the interpretation of our major findings. Additionally, proteinuria was not included as an outcome variable in our study in spite of being included in KDIGO criteria. This was because testing for proteinuria was not routine in the clinic so not all patients in the convenience and research cohorts had such measurements. While evidence exists noting associations of proteinuria with use of ART regimens such as tenofovir [38, 46], studies of adequate duration are limited in low-income countries and more long-term research is required.

Our research was conducted in a region with fewer resources than high-income settings, so findings were reliant on a relatively low number of creatinine results per patient. Furthermore, it is likely that very few tests would have been taken as part of routine testing, instead being taken when a patient is clinically unwell. This would likely add ‘noise’ to study findings. Other possible influencers of eGFR change, such as adherence to ART, or diseases like diabetes hypertension and TB, were not assessed in this study and may have led to confounding.

We included participants from a range of different study cohorts including HEPIK [27], as well as an additional convenience sample specific to this study. This meant that it did not have all the characteristics of a uniform cohort study. In addition, our ability to follow-up participants for additional outcomes such as mortality rates, clinical events or other reasons for loss to follow-up. These data would have been important in generating a clear picture of the real-world effects of ART and HBV status on renal function. We included participants through a variety of studies that did not always share identical inclusion criteria. This may have led to selection bias, in part through over-sampling of HBV-positive participants into our pooled cohort. Lastly, the nature of the study leaves it vulnerable to the effects of confounding, given relatively limited data were available on other comorbidities, co-medications or other factors that can affect eGFR.

## Conclusions

Establishing the most significant contributors towards renal disease in low-income countries is an essential means to be able to minimise its overall health burden [47–49]. Small discrepancies in annual eGFR decline may accumulate over time leading to considerably higher rates of CKD in older populations of PWH. The prevalence of non-communicable diseases such as diabetes and hypertension are increasing in LMICs which will place further burden on the renal health of PWH. Long-term prospective studies encompassing such factors, and exploring simple biomarkers associated with significant eGFR declines, are essential to assess contributors to renal function decline in LMIC populations.

This study is one of the first long-term analyses of the effects of ART and HBV status on renal function in a sub-Saharan African population. This is particularly relevant to populations in West Africa where HBV co-infection is common. The cohort demonstrated an overall increase in eGFR from point of diagnosis to the end of follow-up, though a lack of mortality data complicates the interpretation of this finding. Findings support previous evidence that TDF and protease inhibitors have associations with worsening renal function over time compared with other antiretroviral drugs. Our study highlights the importance that future LMIC-based studies of this kind account for the possibility of initial improvements in eGFR following acute illness. Additionally, studies should include mortality data in their analyses to minimise any effects of survivorship bias.

## Data Availability

The datasets generated and/or analysed during the current study are not publicly available due data restrictions from the source hospital, but are available from the corresponding author on reasonable request.
